# Inequality in Disability in Bangladesh

**DOI:** 10.1371/journal.pone.0103681

**Published:** 2014-07-30

**Authors:** Md. Ismail Tareque, Sharifa Begum, Yasuhiko Saito

**Affiliations:** 1 Department of Population Science and Human Resource Development, University of Rajshahi, Rajshahi, Bangladesh; 2 Population and Health Studies Division, Bangladesh Institute of Development Studies (BIDS), Dhaka, Bangladesh; 3 Advanced Research Institute for the Sciences and Humanities, Nihon University, Tokyo, Japan; 4 School of Medicine, Nihon University, Tokyo, Japan; 5 Duke-NUS Graduate Medical School, Singapore, Singapore; Vanderbilt University, United States of America

## Abstract

**Objective:**

To investigate inequality in disability in Bangladesh.

**Methods:**

The study used both household level and individual level data from a large nationally representative data set, Bangladesh’s Household Income and Expenditure Survey - 2010. Principal component analysis was used to construct a wealth index based on household assets from household level data. Then, using data from 49,809 individuals aged 5 years and over, chi-square tests and logistic regression were performed to test the association between wealth level and disability.

**Findings:**

Women and older people are significantly more likely to report having disabilities than men and younger people. For middle and rich families, respectively, there is a 14 percent lower likelihood of reporting disabilities than for poor families. Changes in the probability of having disabilities are linear with increasing wealth. In addition, the study identifies some significant factors affecting disability, namely, age, sex, education, marital status, and place of residence including divisional differences.

**Conclusion:**

In Bangladesh, worse health among the poor argues for policies prioritizing this group while at the same time giving special attention to women and the elderly.

## Introduction

Socioeconomic inequality in health is a key public health concern [1]. In developed countries, the gradient in the association between socioeconomic status and health is well documented with individuals of higher status living longer, enjoying better health, and experiencing less disability [2]–[6]. Individuals with a lower socioeconomic status generally experience higher morbidity and mortality rates than those with a higher socioeconomic status [7], [8]. Moreover, compared with persons without disabilities, persons with disabilities are reported to have lower educational attainment, lower employment rates, and lower wages when employed, and they are more likely to be income poor [9]–[12].

In developing countries, several studies have also shown that persons with disabilities are poorer than their nondisabled peers in terms of access to education, access to health care, employment, income, social support, and civic involvement. For example, disabled persons had lower school attendance [13]–[19], higher unemployment rates, and lower mean monthly salaries compared with non-disabled persons [13]–[15], [17]–[19]. However, this was not the case in Zimbabwe where the employment rate and mean monthly salaries were not found to be affected by a disability status [16]. Comparing data from household surveys in 13 developing countries, Filmer [20] found that disability is associated with an increased probability of individuals falling in the two poorest quintiles. Using data from 15 developing countries, Mitra, Posarac, and Vick [21] also found that disability was significantly associated with higher multidimensional poverty as well as lower educational attainment, lower employment rates, and higher medical expenditures. Evidence of lower access to health care, education, and the labor market for people with disabilities was also found in Afghanistan and Zambia, but poverty measured by an asset index was not found to be statistically different between people with and without disabilities [22]. Men were found to have a higher rate of disability than women in Zambia [14], but in Viet Nam, men were found to have a lower rate of disability than women [18].

Disability and poverty are reported to have a complex and interdependent relationship [22]. They are intricately linked as both a cause and consequence of each other [23]. The onset of disability may lead to lower living standards and poverty through the adverse impact of disability on education, employment, earnings, and increased expenditures related to disability [21]. Disability, particularly of the family income earner, worsens the position of the family on the income scale, and family members suffer extreme hardship. Moreover, people with disabilities experience poorer levels of health than does the general population [24], and the health care costs for the disabled are a financial catastrophe. There is also the case that people who live in a state of chronic poverty are more likely to have a disability. Chronically poor people are often at risk of ill health and injuries which may lead to disability through a number of routes. They often live in unsanitary and substandard housing conditions, are unable to afford nutritious foods, lack the ability to access clean water and basic sanitation, and are more likely to have unsafe or dangerous jobs. These poverty-related environmental and structural risks for disability mean that the poor who become disabled will descend further into poverty [25]. In developing countries, there is evidence that malnutrition leads to disability [26]. The international development community is beginning to recognize that people with disabilities are disproportionately represented among the poorest and most vulnerable and, thus, that the disabled must be a core issue in development policies and programs [25]. However, to date, there has been no country-wide study in Bangladesh on the type, severity, and causes of disabilities [27]. And, in Bangladesh, there has never been an in-depth analysis of the association between wealth and disability. Thus, to bridge the knowledge and research gaps on the relationship between wealth and health outcomes, this study investigates wealth inequality in disability in Bangladesh. This analysis, using a large nationally representative survey conducted in Bangladesh, takes place in two stages. The first looks at the structure of wealth among Bangladeshi people. The second examines the relationship between wealth and disability.

## Methods

### Data source

This study utilizes data from a large nationally representative survey, Bangladesh’s Household Income and Expenditure Survey (HIES) - 2010, collected by the Bangladesh Bureau of Statistics (BBS), an apex organization of Bangladesh’s Ministry of Planning. The objectives, sampling design, and methodologies are described elsewhere in detail [28]. In brief, the survey provides valuable household level data on household income, expenditures, assets, housing conditions, as well as individual level data on education, employment, health, disability, etc. A two-stage stratified random sampling technique was employed in drawing the sample of HIES - 2010. The data collection was completed in one year (1 February 2010 to 31 January 2011). A total of 12,240 households were selected, with 7,840 from rural areas and 4,400 from urban areas. All individuals from selected households were interviewed for a total of 55,580 individuals; of those, 35,894 came from rural areas and the rest from urban areas.

This study utilizes both household level and individual level data. The wealth index was constructed using household assets from household level data (N = 12,240). Then, wealth index values were assigned to all individuals (N = 55,580) based on household i.d.’s. For the individual level data analyses, we dropped 74 cases with missing values for the wealth index, two cases with missing values for other variables used in the analysis, and restricted our sample to those who were 5 years old and over for a total of 49,809 individuals, of whom 24,555 (49.30 percent) were male and 25,254 (50.70 percent) were female. There are no missing values across disability status in the data set.

### Measures

#### Outcome variable

The International Classification of Functioning, Disability and Health (ICF) developed by the World Health Organization provided a framework for conceptualizing disability. Operationalizing an ICF-based approach to disability has required the development of new measurement tools for use in both censuses and surveys. To date, a short set of six disability-related questions suitable for use in national censuses and surveys has been developed and adopted by the Washington Group. This set of questions, the disability module, is used in HIES –2010 and is consistent with the ICF [29]. It covers six functional domains or basic actions: (1) vision, (2) hearing, (3) walking and climbing, (4) remembering and concentrating, (5) self-care, and (6) speaking and communicating. To assess disabilities in the above six actions, every household member was asked the following questions: (1) Does (name) have difficulty seeing, even if he/she is wearing glasses? (2) Does (name) have difficulty hearing, even if he/she is wearing a hearing aid? (3) Does (name) have difficulty walking or climbing steps? (4) Does (name) have difficulty remembering or concentrating? (5) Does (name) have difficulty with self-care such as washing all over or dressing, feeding, toileting, etc.? And (6) Does (name) have difficulty communicating, for example, understanding or being understood? Each question has four response categories: (a) no difficulty, (b) yes, some difficulty, (c) yes, severe difficulty, or (d) yes, can’t see/hear/walk/remember/self-care/communicate at all. Applying the same cut-off points as in the study for Bangladesh by Tareque, Begum, and Saito [30], the present study categorized having each disability into 2 groups: “no” (no difficulty) with a value of 0 and “yes” (yes, some difficulty/yes, severe difficulty/yes, can’t see/hear/walk/remember/self-care/communicate at all) with a value of 1. And, to create the outcome variable, the disability, i.e. having at least one among the above six disabilities, the six disabilities were combined into one measure with a value of 1 assigned where there was a disability, and a value of 0 assigned otherwise.

#### Independent variables

The independent variables include the wealth index (described in the construction of the wealth index section), age categories (expressed as seven groups: 5–14, 15–24, 25–34, 35–44, 45–54, 55–64, and 65 and over), sex (male and female), two educational categories (illiterate and literate), two marital status categories (married and others, where others includes never married, widowed, divorced, and separated), place of residence (rural and urban), and the divisions (7 divisions, namely, Barisal, Chittagong, Dhaka, Khulna, Rajshahi, Rangpur, and Sylhet). The seven divisions divide Bangladesh into seven major administrative regions. Each division is named after the major city (within the division’s jurisdiction) that serves as the administrative capital of that division.

### Construction of the wealth index

At the household level, the survey includes data on whether or not the household contains the following 27 assets: radio, two-in-one/cassette player, camera/camcorder, bicycle, motorcycle/scooter, motor car, refrigerator/freezer, washing machine, fans, heaters, television, VCR/VCP/DVD, dish antenna/decoder, pressure lamps/petromax, sewing machine, bedroom furniture, drawing room furniture, dining room furniture, carpet, kitchen items - cutlery, kitchen items - crockery, kitchen items - cooking, tube-well, wrist watch/wall clock, mobile, computer/TV card, and boat/others. Each variable (asset) is dichotomized as 1 if present and 0 if not, and the wealth index is constructed using Filmer and Pritchett’s method [31] of employing principal component analysis (PCA). Weights are determined by factor scores derived from the first principal component in the PCA.

The first component is used since it is the one that captures the largest amount of information common to all the items. The first component has an Eigen value of 3.84 capturing fully 14 percent of the variance in the data. Each scoring factor is divided by the sample standard deviation for that asset to yield the final asset weight. And the index is derived by summing the products of the normalized scores for each asset determined by PCA and the final asset weight.

Then, the constructed wealth index values are assigned to each individual based on common variables in both household and individual level data sets. Individuals are then ranked from top to bottom according to the index value as suggested by Rutstein and Johnson [32]. We then establish cutoff values for percentiles of the population, and we refer to the bottom 40 percent as “poor”, the next 40 percent as “middle”, and the top 20 percent as “rich” following Filmer and Pritchett’s study [31]. Several studies suggest that the wealth index is a good alternative to distinguish wealth layers within a population. It is in agreement with measures of household consumption, and it proxies well for other indicators of economic wellbeing [31], [33]–[35]. Compared with expenditure measures, it is reported to be the easier measure of economic status to collect and produces superior, more believable results and equal or greater distinctions in health outcomes [32].

### Analysis plan

We begin by describing study respondents, the structure of assets, and the wealth index. This we accomplish with a descriptive examination of a series of findings that depict attributes of the Bangladeshi population across categories of the wealth index constructed using PCA, as described above. Next, using Chi-square tests, we examine differences in the proportion of disability by wealth categories, sex, and age groups. Then, with a series of logistic regression models we examine associations between the wealth index and disability.

The first model (labeled as Model 1) established a bivariate association by showing results that are unadjusted. The wealth index was treated categorically. Results presented are in the form of odds ratios with confidence intervals and show the odds of having a disability. The next model (Model 2) regressed the outcome variable on wealth index categories, age, and sex. The last model (Model 3) attempted to examine possible mechanisms driving any association by adjusting for additional characteristics such as education, marital status, place of residence, and division. Multicollinearity in the logistic regression analyses in our study was checked by examining the standard errors for the regression coefficients. A standard error larger than 2.0 indicates numerical problems such as multicollinearity among the independent variables [36]. Additionally, a sensitivity analysis was performed considering “no difficulty/yes, some difficulty” as “no disability” and “yes, severe difficulty/yes, can’t see/hear/walk/remember/self-care/communicate at all” as “disabled” for the outcome variable, because research has shown that moderate difficulties (yes, some difficulty) may not be as reliably self-reported as severe difficulties (yes, severe difficulty/yes, can’t see/hear/walk/remember/self-care/communicate at all) [37], [38]. Below, we discuss if the results changed when disability covered only severe difficulties (results not shown).

The net significance of the wealth index in all models was estimated by calculating the difference in the log-likelihood statistic between models that did and did not contain the wealth index (the latter not shown). Finally, the predicted probabilities of reporting a disability were estimated across wealth categories and presented in graph form. To determine the probability, the value of all variables for the final equation, except for wealth categories (Model 3), were held constant, and the mean sample probabilities for the disability were calculated. As such, the result can be interpreted as the probability that an otherwise average respondent would report the disability. The entire statistical analysis of the study was performed with STATA/SE 12.1 (StataCorp LP, College Station, Texas, United States of America).

### Ethical considerations

The Ethics committee at BBS approved a waiver from ethical approval for this retrospective study. As the de-identified data for this study came from secondary sources, this study does not require ethical approval.

## Results

### Sample description

Table 1 provides descriptive information about the sample and the association between selected characteristics and gender with p values of Chi-square tests. The mean age is 29.50 years for males (29.35 years for females), and more than half of the respondents are female. More female respondents are currently married than male respondents. In terms of educational level, a higher percentage of males are literate than females. The urban-rural residence ratio is about 4∶6; and more than one-fourth of respondents come from Dhaka division. Women are significantly more likely to report having at least one disability than men; but when disability covers only severe difficulties, women are less likely to report having a disability than men.

**Table 1:** Characteristics of individuals in sample by gender.

Characteristics	Male (N = 24555)	Female (N = 25254)
**Age (Mean and CI)**	29.50 (29.26–29.74)	29.35 (29.12–29.57)
**Age**		
5–14	28.11	26.27
15–24	19.96	20.44
25–34	15.29	17.92
35–44	13.49	14.41
45–54	10.71	9.64
55–64	6.74	6.09
65+	5.69	5.22
p value	0.000	
**Marital status**		
Currently married	50.13	53.91
Others	49.87	46.09
p value	0.000	
**Education**		
Illiterate	41.45	47.06
Literate	58.55	52.94
p value	0.000	
**Place of residence**		
Urban	36.18	35.36
Rural	63.82	64.64
p value	0.057	
**Division**		
Barisal	7.96	7.99
Chittagong	19.09	19.75
Dhaka	27.98	28.47
Khulna	14.29	13.68
Rajshahi	12.16	11.99
Rangpur	10.32	9.71
Sylhet	8.20	8.39
p value	0.048	
**Having at least one disability**		
No	91.17	89.24
Yes	8.83	10.76
p value	0.000	

p values are of Chi-square tests.

### Structure of wealth and disability

Table 2 provides information about wealth and poverty in two panels. Panel A examines the specific assets owned across the categories of the wealth index. Those living in poor households own just the assets necessary for their livelihood and do not own expensive assets such as cameras, motor cars, refrigerators, computers, etc. Though radios are inexpensive compared with televisions, it seems that Bangladeshi families prefer to have televisions as their source of entertainment rather than radios. Notably, about 95 percent of rich households own televisions, while only 7 percent own radios. Those in the middle category typically own bicycles, fans, televisions, furniture for the bedroom, drawing and dining room furniture, kitchen items, tube-wells, and mobile phones. An appreciative step up in wealth is only evident for the highest category. Individuals in rich households own a fair mix of assets; and a few Bangladeshi households possess a motor car, washing machine, heaters, dish antenna, pressure lamps, and carpet. In sum, the wealth index has efficiently created poor, middle, and rich groups in the absence of direct wealth measures for individual cases.

**Table 2:** Percentage of households owning specific assets, and selected demographic characteristics by categories of wealth index.

	Poor(N = 19629)	Middle(N = 20092)	Rich(N = 10088)	Chi-square
**Panel A: Households owning assets**				
Radio	4.59	5.76	6.78	65.88*
Two-in-one/cassette player	0.43	5.64	22.80	5.2e+03*
Camera/camcorder	0.04	0.53	14.35	5.2e+03*
Bicycle	15.64	30.37	29.75	1.4e+03*
Motorcycle/scooter	0.10	1.81	14.20	4.1e+03*
Motor car	0.00	0.29	2.78	833.69*
Refrigerator or freezer	0.00	2.57	60.28	2.4e+04*
Washing machine	0.01	0.24	1.05	236.95*
Fans	10.28	69.29	96.61	2.4e+04*
Heaters	0.16	0.55	1.97	330.09*
Television	2.40	50.08	94.53	2.5e+04*
VCR/VCP/DVD	0.10	4.03	31.01	9.4e+03*
Dish antenna/decoder	0.05	0.52	3.60	952.44*
Pressure lamps/petromax	0.03	0.12	1.27	361.10*
Sewing machine	1.19	6.99	20.99	3.8e+03*
Bedroom furniture	80.11	90.57	97.00	2.0e+03*
Drawing room furniture	3.34	20.16	66.80	1.5e+04*
Dining room furniture	6.57	15.55	49.86	8.4e+03*
Carpet	0.42	0.80	5.16	1.1e+03*
Kitchen items- cutlery	48.46	60.63	75.59	2.1e+03*
Kitchen items- crockery	49.99	68.41	83.04	3.4e+03*
Kitchen items- cooking	28.94	36.34	50.41	1.3e+03*
Tube-well	33.76	50.40	53.68	1.5e+03*
Wrist watch/wall clock	15.20	54.73	83.20	1.4e+04*
Mobile	32.48	86.86	98.62	1.9e+04*
Computer/TV card	0.11	0.79	12.31	4.1e+03*
Boat/others	5.82	7.01	7.23	31.11*
**Panel B: Demographic characteristics**				
% Female	50.56	50.24	51.89	7.58†
% Currently married	51.55	52.39	52.32	3.20ns
% Literate	38.66	60.17	80.00	4.9e+03*
% Rural	81.74	62.38	33.88	6.7e+03*
**Division**				
Barisal	9.02	7.25	7.40	
Chittagong	14.44	21.39	25.20	
Dhaka	26.84	27.97	31.46	
Khulna	16.17	14.45	8.78	1.7e+03*
Rajshahi	11.34	13.67	10.33	
Rangpur	14.25	8.13	5.52	
Sylhet	7.95	7.14	11.30	
% Having at least one disability	11.02	9.10	8.84	54.76*

Level of significance: * p<0.001; ^†^ p<0.05; ns indicates not statistically significant.

Panel B displays demographic characteristics of the study population across the wealth index. As expected, poor households are made up primarily of illiterate individuals. A slightly higher percent of women exist in rich households than in poor households. Marital status is not significantly associated with the wealth index. A higher percentage of rural households fall in the poor category of the wealth index compared with urban households. A comparatively higher percent of households from Barisal, Khulna, Rajshahi, and Rangpur divisions are in the poor category, and the percent of poor households generally decreases when moving to the rich category of the wealth index. Individuals from poor households have a higher likelihood of having one or more disabilities, and this probability decreases with increases in wealth.

### Association between wealth and disability

Table 3 shows the percent reporting disability by wealth categories, age group, and sex. Across the wealth categories, disability shows an increasing trend with age. Across age groups, with the exception of females aged 15–34, the percent of poor people reporting a disability is higher than that of middle and/or rich people. It is also evident that women have a higher incidence of disability than men across age groups, except in the 5–24 year category.

**Table 3:** Percent of individuals having disability by categories of wealth index, sex, and age groups.

	Male				Female			
	Poor	Middle	Rich	p value	Poor	Middle	Rich	p value
**Age group**								
5–14	3.05	2.03	2.43	0.047	1.83	1.91	2.13	0.820
15–24	3.92	2.40	1.85	0.002	2.68	2.79	1.63	0.090
25–34	4.71	3.51	2.28	0.013	5.89	6.32	4.51	0.149
35–44	9.62	7.79	7.75	0.176	13.74	13.37	11.01	0.171
45–54	17.67	12.75	14.19	0.007	24.95	22.46	20.91	0.178
55–64	26.67	23.60	25.70	0.440	35.66	27.43	27.73	0.003
65+	50.09	40.96	39.78	0.002	57.61	51.84	42.00	0.000
Total (Number)	9705	9997	4853		9924	10095	5235	

p values are of Chi-square tests.

Table 4 presents results of logistic regressions that predict the odds of having a disability among the study population. Model 1 shows that those in the middle wealth category are less likely to report having a disability compared with those in the poor category; and those in the rich category are less likely to report having a disability compared with those in the middle category (keeping middle as the reference category, statistically significant results were obtained; the results are not shown). The advantages of being from a wealthier family for not having a disability are attenuated but persist after incorporating controls for age and sex in Model 2. The effects of age and sex are as expected. The odds of having a disability increase significantly with increases in age. Individuals aged 65 years and over are about 42 times more likely to report having a disability than individuals aged 5–14 years. Females are 1.37 times more likely to have a disability than males.

**Table 4:** Odds ratio for an individual having a disability versus no disability in Bangladesh.

	Model 1		Model 2		Model 3	
	OR	CI	OR	CI	OR	CI
**Wealth index:** Poor (ref)						
Middle	0.81*	0.76–0.86	0.81*	0.75–0.87	0.86*	0.80–0.93
Rich	0.78*	0.72–0.85	0.73*	0.66–0.79	0.86^‡^	0.77–0.94
**Age group:** 5–14 (ref)						
15–24			1.21†	1.02–1.43	1.37*	1.15–1.62
25–34			2.21*	1.90–2.57	2.86*	2.41–3.39
35–44			5.36*	4.67–6.14	6.95*	5.91–8.17
45–54			10.43*	9.12–11.92	13.27*	11.35–15.53
55–64			17.44*	15.19–20.02	21.67*	18.55–25.32
65+			41.99*	36.62–48.16	49.72*	42.85–57.70
**Sex:** Male (ref)						
Female			1.37*	1.29–1.46	1.28*	1.19–1.37
**Education:** Illiterate (ref)						
Literate					0.92^†^	0.85–0.99
**Marital status:** Others (ref)						
Currently married					0.73*	0.67–0.80
**Place of residence:** Urban (ref)						
Rural					1.12^‡^	1.04–1.21
**Division:** Barisal (ref)						
Chittagong					0.91ns	0.78–1.05
Dhaka					1.23^‡^	1.08–1.41
Khulna					1.37*	1.18–1.58
Rajshahi					2.07*	1.79–2.39
Rangpur					1.53*	1.31–1.78
Sylhet					0.90ns	0.76–1.07
**L**	−15953.21		−12819.38		−12667.28	
**Δ-2X LL** a	54.20*		61.41*		16.24*	

OR means odds ratios.

CI indicates confidence interval.

Ref indicates reference category.

Level of significance: * <0.001; ^‡^ <0.01; ^†^ p<0.05; ns indicates not statistically significant.

a Change in −2X Log-likelihood when adding wealth to a model containing other variables. For Model 1, it is the change when adding wealth to a model containing the constant only.

In Model 3, the advantages of being from a wealthier family for not having a disability are further attenuated but persist after incorporating additional controls for education, marital status, place of residence, and division. The study reveals that those in middle and rich families, respectively, there is a 14 percent lower likelihood of reporting disabilities than for poor families. The odds of having a disability by age group are also in the expected direction in Model 3. That is, older people are more likely to have a disability than younger people, but the odds of having a disability increase across age groups in Model 3 (with additional controls) compared with Model 2. The disadvantages of being female for having a disability also persist in Model 3. Compared with uneducated people, educated people are less likely to be disabled in both unadjusted (result not shown) and adjusted models (Model 3). Individuals currently married are significantly less likely to report having a disability than others. Individuals from rural areas are more likely to report having a disability than individuals from urban areas. While people from Sylhet division have the lowest odds of having a disability (not statistically significant), people from Rajshahi division are more likely to have a disability than people from other divisions.

In the sensitivity analysis, a model similar to Model 3 was fitted and, except for sex and division, produced patterns similar to those of Model 3. The people in middle and rich families are less likely to have a disability compared with people in poor families; older people are more likely to have a disability than younger people; educated people are less likely to be disabled than uneducated people; currently married individuals are less likely to report having a disability than others; individuals from rural areas are more likely to have a disability than individuals from urban areas; but women are less likely to have a disability compared with men; and people from Barisal division have higher odds of having a disability than people from other divisions.

Results from Models 1–3 indicate that those in the middle and rich categories are substantially less likely to have a disability than those in the poor category. The Δ-2X LL values are statistically significant for all the models and indicate that the equations showing the relationship between wealth and disability with additional controls are better fitted than the equations without the wealth index.

**Figure 1:**
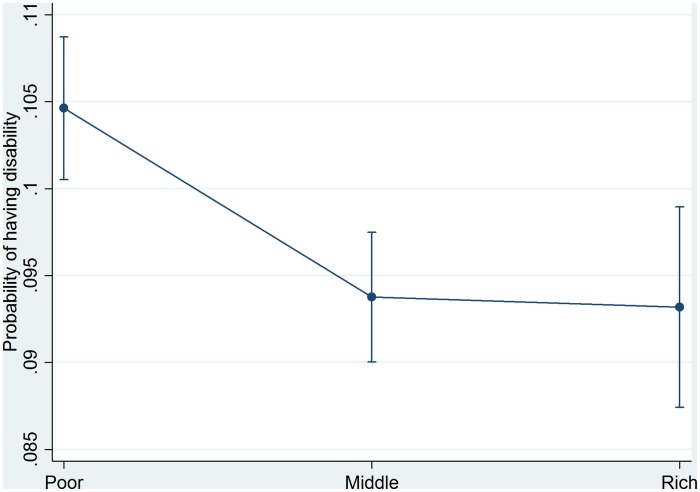
Predicted probabilities of disability by wealth index in Bangladesh.

Predicted probabilities of disability obtained from Model 3 in Table 4 are plotted in Figure 1. The figure emphasizes that those in the middle and rich wealth category have a consistently lower probability of disability.

## Discussion

An abundance of evidence from developed countries links economic wellbeing and health, while only a handful of literature exists for developing countries, and no study examines inequality in disability in Bangladesh. The current study reveals that the disability prevalence was 8.83 percent for males and 10.76 percent for females in 2010. Utilizing data collected in 2003 for the World Health Survey (WHS) for Bangladeshi working-age people (18–65 years), Mitra, Posarac, and Vick [21] reported that the disability prevalence was 9.91 percent for males and 22.90 percent for females. Corresponding figures from HIES-2010 are 9.45 percent for males and 12.05 percent for females. Part of the difference could be due to differences in measures, response categories, and time period. In WHS, there were five response categories - 1: no difficulty, 2: mild difficulty, 3: moderate difficulty, 4: severe difficulty, and 5: extreme difficulty/unable to do. The WHS-based disability measures may underestimate disability prevalence, because they do not cover difficulties in hearing and communicating. They may also overestimate disability prevalence, because the introduction to the section containing questions on difficulties in functioning does not explain, as does the introduction to the questions of the Washington Group, that reported limitations or restrictions need to be related to a “health problem.”

The present study provides some notable results in relation to the test of the relationship between wealth index and disability. The wealth index, a more permanent indicator than income or consumption [32], separates the study population into three groups. The bottom 40 percent consists of households that own a few assets, while the next 40 percent, labeled as middle, own more assets. Undoubtedly, those in the highest quintile, labeled as rich, do appear to be qualitatively better off compared with the rest of the population. The following are some conclusions regarding the above wealth categories and disability: (a) Women are significantly more likely to report having a disability than men. (b) There is a differential in disability across gradients of wealth. A consistent significant decline in having a disability was identified when moving from the poor to the rich category. (c) Changes in the probability of having a disability are linear with increasing wealth (Figure 1). Each gradient increase in wealth relates to a noticeably lower probability of having a disability. Moreover, the current study identifies some significant factors that affect disability, namely, age, sex, education, marital status, and place of residence including divisional differences.

Women reported suffering more from disability than men. Mitra and Sambamoorthi [39] also found similar results of higher disability prevalence among women than men for Bangladesh. In Viet Nam, women were also found to have a higher incidence of disability than men [18]. However, the sensitivity analysis revealed the opposite scenario that women are less likely to have a disability than men. This is because a higher percentage of women than men reported having moderate disability. Part of the reason for this higher incidence of disability among women could be the less than adequate care and services for pregnant and delivering mothers. These mothers, while performing their reproductive role, probably encounter some lasting health problems which for cultural reasons remain undisclosed and make them fall frequently sick. Today, obstetric fistula is largely confined to the poor of tropical cultures [40]. It could be a reason for the higher incidence of disability among women than men in Bangladesh.

Poor nutritional status is also a key health problem in Bangladesh. Young children and women of reproductive age are especially vulnerable to nutritional deficits and micronutrient deficiencies. Overall, 65 percent of ever-married women age 15–49 years live in a food secure environment, and only 35 percent of women in the lowest wealth quintile are food secure compared with 90 percent of women in the highest wealth quintile [41]. Malnutrition in adults results in reduced productivity, increased susceptibility to infections, slow recovery from illness, and, for women, an increased risk of adverse pregnancy outcomes [42]. In addition, the lower status of women in Bangladesh deprives them of many basic things in life including food, nutrition, health care, a secure life, a respectable living, mental peace, an abuse-free life, etc. This, in turn, may contribute to their bad health. The female disadvantage in disability in this study needs special attention.

In developed countries, worse health among the most socially disadvantaged exists, and excess mortality, in large part the result of poor health, is also found to be associated with lower income [6], [43]. An aging study of Cambodia shows that the socially disadvantaged have the most health problems [33]. In line with other studies [20], [21], the current study shows that the most socially disadvantaged, i.e. poor people, have a higher incidence of disability than middle and/or rich people.

Assuming that the association runs from wealth to disability, it is quite possible that a series of demographic factors intervene. The current study shows a negative association between age and disability, with older individuals more likely to report disability than their younger counterparts. Mitra and Sambamoorthi [39] also found that older people in Bangladesh had a higher disability prevalence than their younger counterparts. Health problems, the types of disability used in this study, usually increase with increasing age and, thus, it is not surprising to have the highest odds ratios of having a disability for those aged 65+. The above result could be partly explained by aging studies that reveal that older individuals are more likely to report poor health [44], [45] and disability [46] than their younger counterparts. And, therefore, our study urges policy makers to pay special attention to the elderly when making policies regarding disabilities.

Educated persons may earn more, adopt healthier lifestyles, and, consequently, have less disability. In the United States, poor and poorly educated people were found to die at higher rates than those with higher incomes or better education [8]. In Japan, education has only a small effect on disability, and the robust education-health relationship found in Western societies does not seem applicable in Japan [47]. In our study, education is found to have a positive influence on disability in both the final adjusted model and the sensitivity analysis. Healthy life partners are usually chosen for marriage; therefore, currently married individuals might have less disability than others. In the present study, those who are currently married are less likely to report having a disability than others. Regional differences in disability are also of interest. Persons from places with insufficient transportation, treatment, and health care facilities may have more disabilities. In conformity with a study conducted by the Danish Bilharziasis Laboratory for the World Bank [27], we also observe that in Bangladesh most people with disabilities live in rural areas. In terms of divisional differences of disability, we also found a higher prevalence of disability in Rajshahi division than in other divisions, while the sensitivity analysis showed a higher prevalence of disability in Barisal division. Therefore, this study suggests implementing disability-related policies that pay particular attention to Rajshahi division and target rural areas throughout Bangladesh.

### Limitations

This study’s limitations should be considered when interpreting our findings. First, the data are cross-sectional. We did not control for possible endogeneity of wealth: are you poor because you are disabled and cannot work? Or, are you disabled because being poor you were not able to access health services and get adequate care? Second, the data are self-reported. Although this could be a possible source of bias, studies have shown that self-reported data on functional disability were consistent with medical diagnoses [48]. Third, structural and functional aspects of human body more than capacity and/or performance were included for quantifying disability. Despite this limitation, this most recent and reliable nationally representative large data set presents a clear scenario of wealth inequality in disability among Bangladeshis. What is certainly needed to address the above limitations is a health transition analysis through routine disability reporting with wealth inequality. This is where a longitudinal study could play a vital role.

## Conclusions

In summary, as a first study examining economic inequality and its association with disability in Bangladesh, this study has provided some notable results. In particular, a gradient in disability was found across wealth categories; individuals from middle/rich families are shown to have less disability than those living in poor families. Also, the prevalence of disabilities among older people and women is greater than among their younger and male counterparts, respectively. Therefore, in Bangladesh, worse health among the poor argues for policies prioritizing this group while at the same time giving special attention to women and the elderly.
